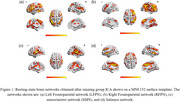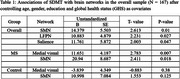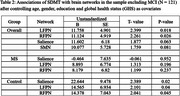# Mild Cognitive Impairment status changes the relationship between Functional Connectivity and Processing Speed in a sample of older adults with and without MS

**DOI:** 10.1002/alz70857_107104

**Published:** 2025-12-26

**Authors:** Siddharth Nayak, Mark E. Wagshul, Roee Holtzer

**Affiliations:** ^1^ Albert Einstein College of Medicine, New York, NY, USA; ^2^ Albert Einstein College of Medicine, Bronx, NY, USA

## Abstract

**Background:**

Human neuroimaging efforts to understand the pathophysiology of Alzheimer's disease have recently shifted to pre‐clinical AD stage, that is, the conversion period from cognitively normal aging to Mild cognitive impairment (MCI) status. Thus, it becomes crucial to characterize the changes associated with and without the presence of MCI when considering brain – cognitive associations.

**Method:**

The initial dataset includes 167 older (66.53 ± 6.19 years) adults (82 MS and 85 controls). In the overall sample, 121 participants were diagnosed as cognitively normal, and 46 participants were diagnosed with mild cognitive impairment (MCI). All the participants were imaged for resting‐state functional magnetic resonance imaging (rsfMRI) and structural MRI scans. We employed a data‐driven approach, Group Independent Component Analysis (ICA) to characterize brain networks in the whole sample and then derive subject level network expressions by dual regression. Symbol Digit Modalities Test (SDMT) was used as a primary outcome measure for processing speed. Multiple linear regressions were used to predict SDMT from brain network expression scores.

**Result:**

LFPN, RFPN, SMN, and salience networks are visualized on the surface of the brain as shown in Figure 1. In the overall sample, SMN (*p* = 0.01), LFPN (*p* = 0.027), and salience (*p* = 0.047) networks showed associations with SDMT. In the stratified analysis, SMN (*p* = 0.018) and medial visual (*p* = 0.007) networks showed association with SDMT in the MS group while there were no significant associations in the control group as shown in Table 1.

When we removed participants with MCI status from the overall sample, LFPN (*p* = 0.018), and RFPN (*p* = 0.026) networks showed associations with SDMT. In the stratified analysis, RFPN (*p* = 0.045), LFPN (*p* = 0.04) and salience (*p* = 0.02) networks showed association with SDMT in the control group while there were no significant associations in the MS group as shown in Table 2.

**Conclusion:**

Our results provide evidence of the role of MCI status impacting brain – cognitive associations in older adults with and without MS. In addition, the findings throw light on the dynamic patterns of functional connectivity in pre‐clinical AD stage.